# A Systematic Review, Meta-Analysis, and Meta-Regression Evaluating the Efficacy and Mechanisms of Action of Probiotics and Synbiotics in the Prevention of Surgical Site Infections and Surgery-Related Complications

**DOI:** 10.3390/jcm7120556

**Published:** 2018-12-16

**Authors:** Karolina Skonieczna-Żydecka, Mariusz Kaczmarczyk, Igor Łoniewski, Luis F. Lara, Anastasios Koulaouzidis, Agata Misera, Dominika Maciejewska, Wojciech Marlicz

**Affiliations:** 1 Department of Biochemistry and Human Nutrition, Pomeranian Medical University, Szczecin 71-460, Poland; karzyd@pum.edu.pl (K.S.-Ż.); sanprobi@sanprobi.pl (I.Ł.); dmaciejewska.pum@gmail.com (D.M.); 2 Department of Clinical and Molecular Biochemistry, Pomeranian Medical University, Szczecin 70-111, Poland; mariush@pum.edu.pl; 3 Division of Gastroenterology, Hepatology, and Nutrition, The Ohio State University Wexner Medical Center, Columbus, OH 43210, USA; Luis.Lara@osumc.edu; 4 Centre for Liver & Digestive Disorders, Royal Infirmary of Edinburgh, Edinburgh EH16 4SA, UK; akoulaouzidis@hotmail.com; 5 Department of Child and Adolescent Psychiatry, Charité Universitätsmedizin, Berlin 13353, Germany; agata.misera@charite.de; 6 Department of Gastroenterology, Pomeranian Medical University, Szczecin 71-252, Poland

**Keywords:** surgical site infections (SSIs), probiotics, prebiotics, synbiotics, surgery, adverse events, microbiota, meta-analysis, systematic review

## Abstract

Intestinal microbiota play an important role in the pathogenesis of surgical site infections (SSIs) and other surgery-related complications (SRCs). Probiotics and synbiotics were found to lower the risk of surgical infections and other surgery-related adverse events. We systematically reviewed the approach based on the administration of probiotics and synbiotics to diminish SSIs/SRCs rates in patients undergoing various surgical treatments and to determine the mechanisms responsible for their effectiveness. A systematic literature search in PubMed/MEDLINE/Cochrane Central Register of Controlled Trials from the inception of databases to June 2018 for trials in patients undergoing surgery supplemented with pre/pro/synbiotics and randomized to the intervention versus placebo/no treatment and reporting on primarily: (i) putative mechanisms of probiotic/symbiotic action, and secondarily (ii) SSIs and SRCs outcomes. Random-effect model meta-analysis and meta-regression analysis of outcomes was done. Thirty-five trials comprising 3028 adult patients were included; interventions were probiotics (*n* = 16) and synbiotics (*n* = 19 trials). We found that C-reactive protein (CRP) and Interleukin-6 (IL-6) were significantly decreased (SMD: −0.40, 95% CI [−0.79, −0.02], *p* = 0.041; SMD: −0.41, 95% CI [−0.70, −0.02], *p* = 0.006, respectively) while concentration of acetic, butyric, and propionic acids were elevated in patients supplemented with probiotics (SMD: 1.78, 95% CI [0.80, 2.76], *p* = 0.0004; SMD: 0.67, 95% CI [0.37, −0.97], *p* = 0.00001; SMD: 0.46, 95% CI [0.18, 0.73], *p* = 0.001, respectively). Meta-analysis confirmed that pro- and synbiotics supplementation was associated with significant reduction in the incidence of SRCs including abdominal distention, diarrhea, pneumonia, sepsis, surgery site infection (including superficial incisional), and urinary tract infection, as well as the duration of antibiotic therapy, duration of postoperative pyrexia, time of fluid introduction, solid diet, and duration of hospital stay (*p* < 0.05). Probiotics and synbiotics administration counteract SSIs/SRCs via modulating gut-immune response and production of short chain fatty acids.

## 1. Introduction

One of the most challenging health care issues worldwide are surgical site infections (SSIs) [1,2]. Timely administration of effective preoperative antibiotics along with other perioperative quality control interventions recommended by various guidelines [3,4,5] have resulted in a significant reduction of the rate of SSIs. Despite these efforts, globally SSIs occur in 9–22% of procedures, with a direct correlation with the human developmental index [1]. SSIs result in prolonged hospitalizations, unscheduled re-admissions, extended duration of antibiotic therapy, increase mortality rate, and pose high costs to healthcare systems. Therefore, it is of priority to look for other effective, evidence-based interventions capable of reducing the incidence of life-threatening SSIs [6,7,8].

There is increasing evidence that human intestinal microbiota play an important role in the pathogenesis of SSIs. Although historically, gut flora has been considered as a pathogen in human infections [9], recent studies show that alteration of the human microbiome (dysbiosis) may play a role in the pathogenesis of SSIs and other surgery-related complications (SRCs) [10,11,12]. Human gut microbiota composition fluctuates on a daily basis depending predominantly on diet, but also exercise, medications, and exposure to stressful events [13,14,15,16]. The general health status of a patient scheduled for surgery is of particular interest, and the make-up of the microbiota could be of particular interest, because it is believed that the majority of hospital infections originate from the patient’s own microbiota, in part due to noxious and stressful surgical preparatory procedures [2]. Supporting the role of microbiota, it has been shown that mechanic bowel preparation (MBP) before gut resection, accompanied by oral antibiotic therapy, reduces the number of infectious complications, including anastomotic leakages by almost half [17]. However, multiple studies have reported vast disturbances in microbial counts and diversity following these procedures that may itself create microbiota disturbances with health consequences [18,19]. 

The surgical procedure itself and other pathology not even related to the gastrointestinal tract may be a major cause of alterations in the intestinal microbiota. There are numerous examples in the literature. Dysbiosis has been described in the excluded colon after small bowel stoma [20]. Major burn injury was described to reduce two major phyla within the human gut and to increase *Gammaproteobacteria* class involved in SSIs [21]. Significant changes of gut flora with increased virulent *Escherichia coli*, *Pseudomonas aeruginosa*, and *Enterococcus faecalis* counts have been described with surgical procedures [21,22,23]. Surgical reconstructions of the gastrointestinal (GI) tract may delay the microbiota refaunation [24,25], and result in enhanced virulent phenotype expression [26]. In severe injuries, more virulent pathogens may predominate in the intestinal ecosystem [27], disrupt the intestinal barrier structure and function, which facilitates the bacterial translocation, and may result in SSIs.

It thus appears that manipulating gut microbiota composition to a healthier variety could be promising. Administration of beneficial microbes (probiotics), fiber (prebiotics), or both (synbiotics) could be an attractive strategy to diminish the incidence of SSIs [28]. There are randomized, double-blind, placebo-controlled trials and meta-analyses that support the efficacy of this strategy [28,29,30,31,32,33]. A recently published meta-analysis aimed to find evidence on prebiotics, probiotics, and synbiotics supplementation on postoperative complications (mostly infective) in surgical patients [28,29,32,34]. Additionally, Wu et al. [29] estimated the efficacy of probiotics and antibiotics combination in the prevention of SSIs and the decrease of antibiotics usage in colorectal surgery, and Kasatpibal et al. [28] conducted a network meta-analysis (NMA) to evaluate the efficacy of probiotics, prebiotics, and synbiotics in reducing SSIs as well as other postoperative complications. Although probiotics have already been used as prophylaxis against SSIs, to the best of our knowledge, none of the guidelines recommend their use. Among the reasons could be lack of data on the precise mechanisms of such interventions in lowering the risk of SSIs and the fact that studies aimed at elucidating the effect of probiotic action on mucosal and stool microbiota lack correlation with clinical outcomes [35]. 

Therefore, this systematic review was performed to study the role of probiotics and synbiotics in the prevention of SSIs and SRCs. In particular, our study aimed to evaluate:

- The mechanism of action of probiotics and synbiotics in prevention of SSIs;
- The influence of probiotics on gut microbiota alterations related to the surgery;
- A possibility to establish recommendations concerning strain(s), dose, and mode of administration of probiotic in the prevention of SSI and SRCs.

A random-effect model meta-analysis to determine putative mechanisms associated with such intervention was also performed. The meta-analysis (MA) evaluated all available data on the usefulness of probiotics in the prevention of SSIs/SRCs in patients undergoing abdominal surgery. The findings could result in a call to determine the appropriateness of implementation probiotics into clinical practice and consideration for inclusion in guidelines as a potentially cost-effective and life-saving therapy. Finally, a meta-regression was performed in order to try to identify a particular probiotic strain of formula, dose, and duration of the probiotic supplementation, which could be recommended as treatment to prevent SSIs.

## 2. Materials and Methods

### 2.1. Search Strategy and Inclusion Criteria

Two independent authors (K.S.-Z., M.K.) searched PubMed/MEDLINE/Cochrane Central Register of Controlled Trials from the inception of databases until 1 June 2018 in English for human trials assessing the efficacy of pre/pro/synbiotic administration in reducing the incidence of SSIs and SRCs. The following search terms with medical subject headings (MeSH–**bold font**) Supplementary Concept Record terms (SCR *italic font*) and free text terms were used: (“**probiotics**” OR probiotic * OR “**prebiotics**” OR symbiotic * OR fiber OR “**dietary fiber**” OR microbiota *) AND (operation OR “surgical procedure” OR “**surgical procedures, operative**” OR “**general surgery**” OR surgery OR **transplantation** OR “surgical operation” OR surgery OR “abdominal surgery” OR “colorectal surgery” OR “colectomy” OR “small bowel surgery” OR **hepatectomy** OR “biliary surgery” OR “pancreas surgery” OR proctology * OR proctocolonic surgery * OR intestine surgery *) AND (readmission OR “readmission rate” OR **mortality** OR **morbidity** OR **sepsis** OR procalcitonin OR **calcitonin** OR leakage OR “surgical infection” OR “surgery site infection” OR leakage OR “anastomotic leakage” OR SSI OR post-operative wound infection * OR postoperative wound infection * OR complication OR **peritonitis** OR **abscess** OR translocation OR **lactulose** OR *zonulin* OR calprotectin OR **ileus** OR “postoperative ileus”). Apart from the electronic search, a manual review of reference lists from existing meta-analysies and relevant reviews was performed.

We used the following inclusion criteria:

1. treatment with pro-/pre-/synbiotics;
2. randomisation to pre/pro/synbiotic versus placebo/monotherapy/standard care; and
3. available meta-analyzable endpoint/change score data on outcomes placed below.
4. if a study contained more than two arms, the data were abstracted separately for each comparator.

### 2.2. Data Abstraction

Two authors (K.S.-Z., M.K.) independently, in accordance with the Preferred Reporting Items for Systematic Reviews and Meta-Analyses (PRISMA) [36], abstracted information from each study, including details of the study (e.g., study design, treatment protocol, duration, number of subjects, gut barrier and SRCs parameters, and risk of bias), intervention (e.g., pre/pro/symbiotic, agent name, dosage, and duration of treatment), and primary patient characteristics (e.g., age, sex, and reason for the surgery). In case of missing data, a request letter for additional information was sent to authors. Any inconsistencies were referred by the senior author (W.M.). 

### 2.3. Outcomes

The primary outcomes that were extracted from each study were the gut-related parameters associated with the putative mechanism of pre/pro/symbiotic action: bacterial translocation, lactulose/mannitol ratio, short chain fatty acids production, zonulin, calprotectin, gut microbiota composition, diamine oxidase (DAO) activity, as well as non-specific indices of inflammation such as C-reactive protein (CRP), interleukin-6 (IL-6) plasma concentration and white blood cells (WBC) count. To update the data reported by other authors on the effectiveness of pre/pro/synbiotics evaluating such interventions in the prevention of SSIs/SRCs the following secondary outcomes were evaluated: abdominal distention, anastomotic leakage, diarrhea, intraabdominal abscess, mortality, methicilin resistant *staphylococcus aureus* infection, peritonitis, pneumonia, re-operation, sepsis, SSIs, superficial incisional SSIs, deep organ/space SSIs, urinary tract infections, blood loss, duration of antibiotic therapy, duration of postoperative pyrexia, the time of implementation of fluid and solid diet, hospital and intensive care unit stay duration, and operating time. 

### 2.4. Data Synthesis and Statistical Analysis

A random effects meta-analysis [37] of outcomes for which at least three studies contributed data was conducted using software (Comprehensive Meta-Analysis, version 3.3.070; http://www.meta-analysis.com). The between-study variance (τ^2^) was estimated using the method of moments (DerSimonian and Laird) and the assumption of homogeneity in effects was tested using the Q statistic with a k-1 degree of freedom (k—the number of studies). Pooled standardized mean difference (SMD) in change score/endpoint scores was used to analyze group differences in case of continuous variables. For nominal outcomes the summary risk ratio (RR) was calculated. A two-tailed Z test was used to test the null hypothesis that the summary effect is zero. In addition to classical meta-analysis, a meta-regression was performed under the random-effects model for both continuous and nominal study level covariates. The regression models with single covariates were fit. Funnel plots were inspected to quantify whether publication bias could have influenced the results. The Egger’s regression intercept test for asymmetry of the funnel plots was used. The statistical significance was adopted at two-side *p* value < 0.05. 

### 2.5. Risk of Bias

Two authors (K.S.-Z. and M.K.) independently assessed the risk of bias using the Cochrane Collaboration’s tool for assessing risk of bias [38]. When a discrepancy occurred, a third author (I.Ł.) was involved. The quality of a study was reported as high when there were more than three low risk of bias assessments. 

## 3. Results

### 3.1. Search Results

**Figure 1:**
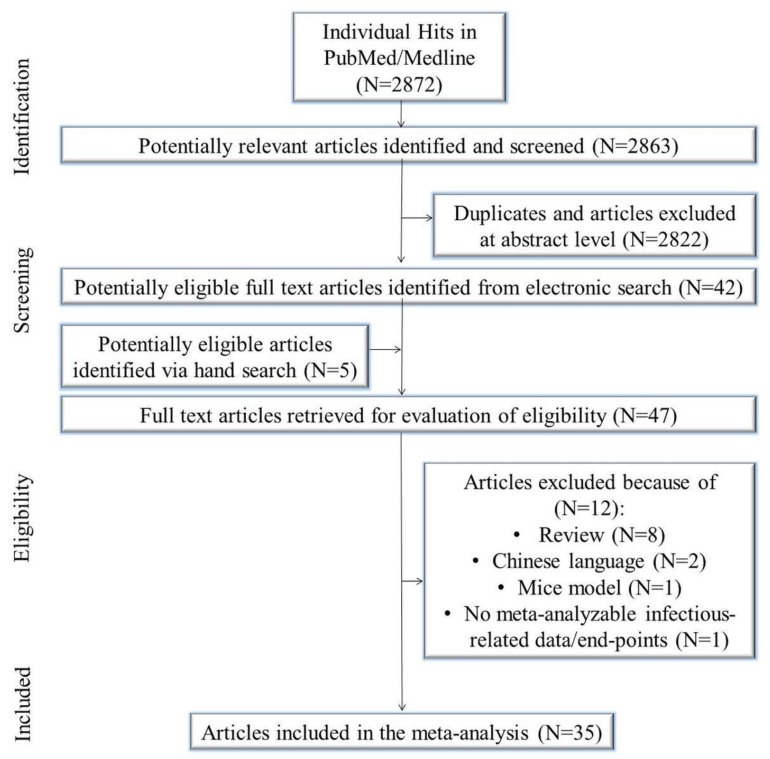
Study flow chart.

The initial search yielded 2872 citations. Of these, 2822 were duplicates and/or removed after title/abstract evaluation. Five manuscripts were identified using a manual search. Forty-seven articles underwent a full-text review, and some were excluded because they were reviews/meta-analysis/systematic review (*N* = 8), in the Chinese language (*N* = 2), mice model (*N* = 1), and contained no meta-analyzable infectious related data/end-points (*N* = 1). Eventually, 35 studies were included in the meta-analysis [39,40,41,42,43,44,45,46,47,48,49,50,51,52,53,54,55,56,57,58,59,60,61,62,63,64,65,66,67,68,69,70,71,72,73] (Figure 1).

### 3.2. Study, Patient and Treatment Characteristics

Of the 35 studies included, the majority were double-blind trials (*N* = 17) [39,42,45,46,47,49,50,51,52,56,60,61,64,71,72,73,74]. The mean study duration was 14.5 ± 5.58 (range: 3–28) days. In 16 studies [39,41,42,46,49,50,51,52,53,54,56,63,64,65,69,70], probiotic intervention was used, while synbiotics were administered in 19 trials [40,43,44,45,47,48,55,57,58,59,60,61,62,66,67,68,71,72,73]. There were two major groups per surgery performed: hepatopancreatobillary (*N* = 15) [40,43,46,51,54,58,63,64,66,67,68,70,71,72,73] and colorectal (*N* = 11) [31,41,47,49,50,51,52,56,61,62,65]. In seven studies [42,44,45,48,53,55,60] the procedure was not specified. Two trials involved oesophagectomy [57,59]. The most commonly utilized comparator was placebo (*N* = 15) [31,42,43,45,47,49,50,51,52,56,60,63,64,69,70]. There were 3028 patients included, with a male predominance (*n* = 1748, 57.73%). Details are given in Table 1.

### 3.3. Microbiota and Putative Mechanism of Probiotic/Synbiotics’ Action in SSIs/SRCs Prevention—Primary Outcomes

Gut microbiota analyses were present in 14 studies [40,41,42,43,44,52,55,56,57,58,59,64,65,67]. The results confirmed postoperative microbiome alterations in study groups compared to controls. Most studies identified *Lactobacillus* (phylum *Firmicutes*) and *Bifidobacterium* (phylum *Actinobacteria*) as beneficial for the outcomes. Nine studies [40,41,42,55,56,57,59,67] reported elevations in *Bifidobacterium* genus (or its particular species) including patients supplemented with microbial agents, but did not reach statistical significance for a benefit. *Lactobacillus* concentrations were elevated post-surgery in six studies [40,57,59,64,67,75]. In contrast, decreased numbers of beneficial microbes and increased abundance of harmful species (*Enterobacteriaceae, Pseudomonas*, *Staphylococcus*, and *Candida*) were reported in a few no-intervention groups [40,42,44,57]. One study [56] reported a *Bifidobacterium/E. coli* ratio. In two studies [43,58], there were no significant differences in bacterial species abundance between the groups. For example, Usami et al. [58] concluded that two weeks after the surgery microbiota composition resembled that of before the surgery regardless of the intervention. However, changes of fecal microbiota composition observed by Usami et al. [58] were not consistent with results reported by other authors [67]. Reasons for this discrepancy might be associated with the difference in intestinal microbiota between liver cirrhosis and biliary surgery patients and/or no administration of enteral nutrition in their study [40,67]. Details are given in Table 2.

**Figure 2:**
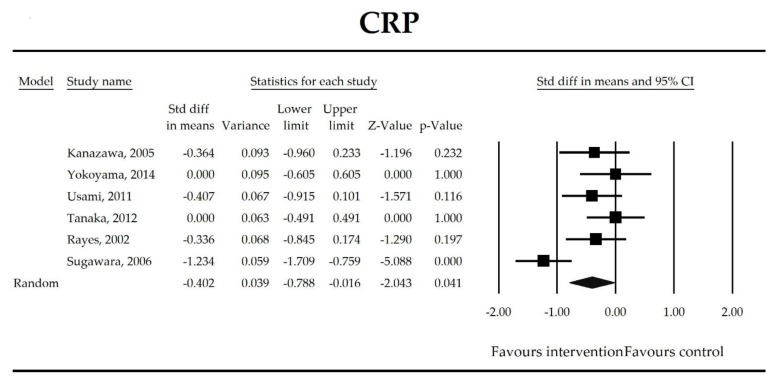
The effect size (standardized mean difference) for the concentration of CRP in patients taking probiotics (intervention) vs. no probiotics (control).

**Figure 3:**
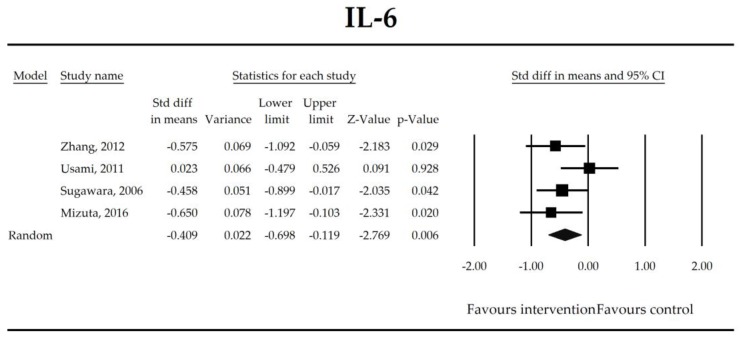
The effect size (standardized mean difference) for the concentration of IL-6 in patients taking probiotics (intervention) vs. no probiotics (control).

**Figure 4:**
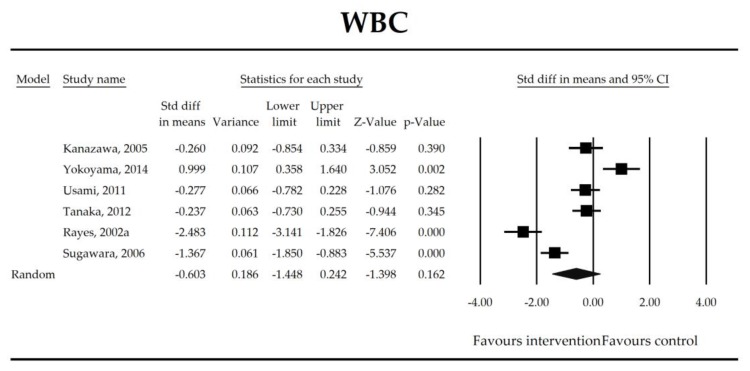
The effect size (standardized mean difference) for the concentration of WBC in patients taking probiotics (intervention) vs. No probiotics (control).

**Figure 5:**
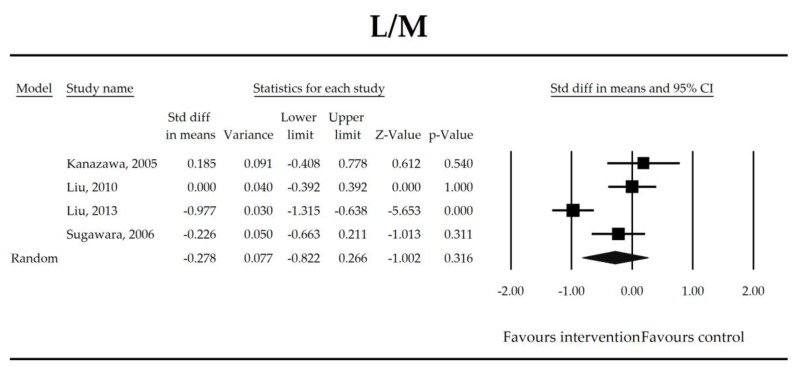
The effect size (standardized mean difference) for the lactulose/mannitol (L/M) ratio in patients taking probiotics (intervention) vs. no probiotics (control).

**Figure 6:**
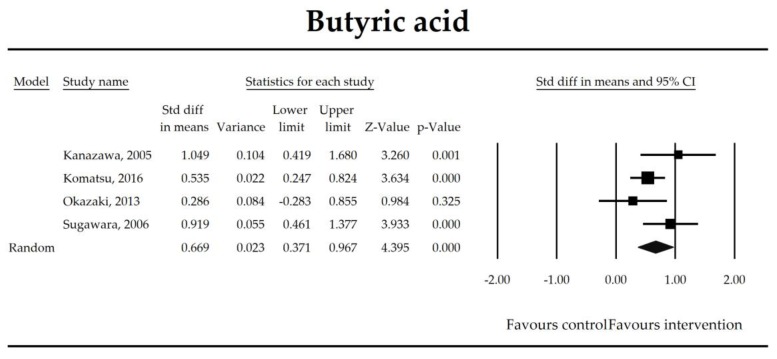
The effect size (standardized mean difference) for the concentration of butyrate ratio in patients taking probiotics (intervention) vs. no probiotics (control).

**Figure 7:**
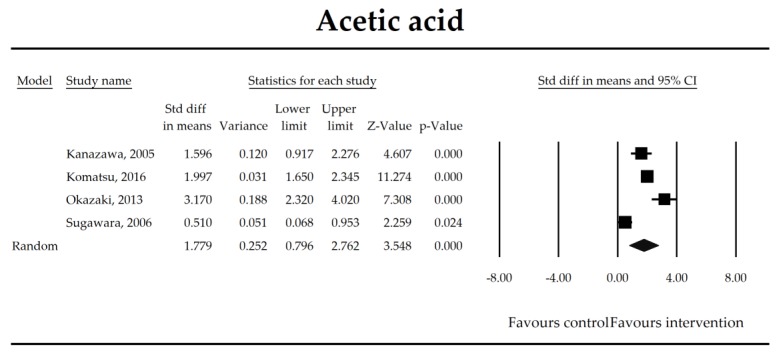
The effect size (standardized mean difference) for the concentration of acetic ratio in patients taking probiotics (intervention) vs. no probiotics (control).

**Figure 8:**
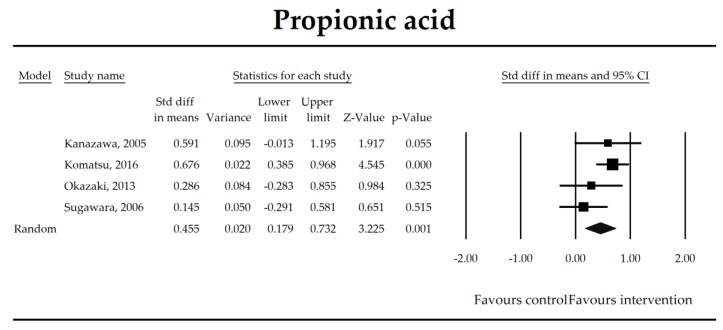
The effect size (standardized mean difference) for the concentration of propionic ratio in patients taking probiotics (intervention) vs. no probiotics (control).

**Figure 9:**
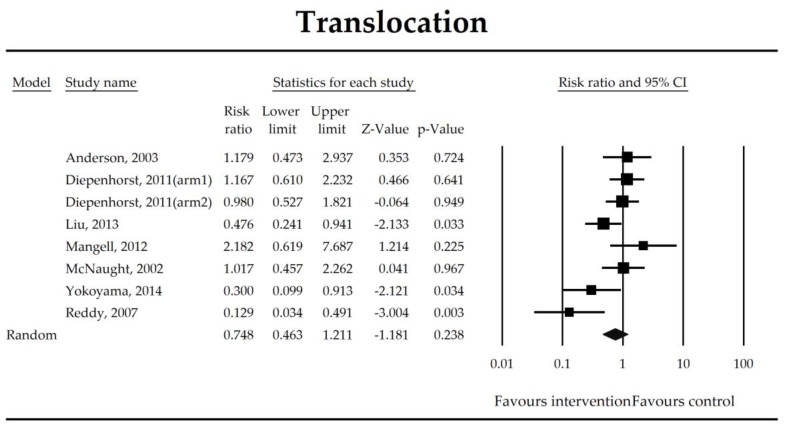
The effect size (risk ratio) for the overall effects of probiotics in the prevention of bacterial translocation.

Putatively factors associated with the mechanism of pro/synbiotic action were searched with a focus on gut barrier integrity. These included: (i) bacterial translocation, (ii) lactulose/mannitol permeability test, and (iii) short chain fatty acids (butyrate, acetate, propionate) concentration, as well as non-specific markers of inflammation: (iv) C-reactive protein, (v) IL-6, and (vi) WBC counts. Diamine oxidase (DAO) activity was analyzed in two studies only [40,58], therefore excluded from metanalysis. CRP and IL-6 were significantly decreased (SMD: −0.40, 95% CI [−0.79, −0.02], *p* = 0.041; SMD: −0.41, 95% CI [−0.70, −0.12], *p* = 0.006, respectively) and short chain fatty acids (SCFAs)–acetic, butyric and propionic acids–were elevated (SMD: 1.78, 95% CI [0.80, 2.76], *p* = 0.0004; SMD: 0.67, 95% CI [0.37, 0.97], *p* = 0.00001; SMD: 0.46, 95% CI [0.18, 0.73], *p* = 0.001, respectively) in patients supplemented with probiotics. No other statistically significant results were found. Results are presented in Table 3 and Figure 2, Figure 3, Figure 4, Figure 5, Figure 6, Figure 7, Figure 8 and Figure 9.

### 3.4. Surgery Related Complications (SRCs) and Secondary Outcomes

To evaluate the effectiveness of pro/synbiotic interventions in reducing the incidence of SSIs/SRCs, data was extracted from common surgery-related clinical outcomes. Consequently, meta-analyses were conducted on parameters reported in at least three studies and the data confirmed that microbial supplementation was associated with a significant reduction in the incidence of SSIs and SRCs including: (i) abdominal distention, (ii) diarrhea, (iii) pneumonia, (iv) sepsis, (v) superficial incisional infection, (vi) urinary tract infection, (vii) duration of antibiotic therapy, (viii) duration of postoperative pyrexia, (ix) time of fluid introduction and (x) solid diet, and (xi) duration of hospital stay. Data are given in Supplementary Table S1. Representative forest plots of secondary outcomes are presented in Supplementary Figures S1 and S2. Other forest plots are available upon request.

To obtain data useful for drawing clinical recommendations and new guidelines a meta-regression was conducted (Table 3). Based on the analysis of the selected studies, it was not possible to find a particular probiotic formula or strain, its dose or duration of the probiotic supplementation that could be recommended to manage either primary or secondary outcomes analyzed in this study (*p* > 0.05). An inverse correlation was only found for propionic acid concentration. For every increase of one unit (day) in treatment duration, the SDM for propionate decreased by 0.0355 (*p* = 0.049). Also effect sizes were found to be independent of the timing of the intervention (pre + post vs. only post-surgery). It was not possible to show whether the quality of the trial could have influenced its results (*p* > 0.05). 

### 3.5. Risk of Bias

An analysis of the overall risk of bias from the studies included in the meta-analysis was limited by restricted information being provided. For example, random sequence generation bias could not be determined in 15 studies and allocation concealment bias could not be studied in 13 papers. The unclear risk of bias in performance, detection, short-term outcomes, and reporting sections were reported in 9, 11, 3, and 12 studies, respectively. It was not possible to determine other risks of bias in 24 papers. Overall, 14 studies were of high quality and 21 of low quality. One study achieved maximum points of low risk assessments (i.e., 7 points) and only two studies achieved no low risk of bias assessments points (i.e., 0 points). The results are in Table S2 (Supplementary Material).

## 4. Discussion

To the best of our knowledge this meta-analysis of 35 trials and 3028 patients is the first one to exclusively investigate the effect and possible mechanism of action of pro-/synbiotics to lower the risk of SSIs and SRCs. The study shows that microbial agents administered perioperatively have the potential to increase the abundance of beneficial bacteria within the gut, elevate the synthesis of short chain fatty acids and thus reduce the immune response. Consequently, it appears to indicate that pro-/synbiotics may serve as preventive strategy toward SSIs and SRCs.

The data are mounting that the host complex of bacteria, fungi, viruses, and *Archaea* contribute to human biology [76]. In patients scheduled for elective abdominal surgery, the gut microbiota might undergo alterations that have an impact on surgery outcomes. In this study in patients not treated with any microbial agents perioperatively, the predominance of beneficial microbes was decreased, but the counts of potentially harmful ones were elevated. Eubiosis and a proper abundance of protective bacteria in the gut may protect the host against pathogens [75]. In this meta-analysis, the majority of the studies showed that pro-/synbiotic treatment reduced the number of *Enterobacteriaceae*. However, Mangel et al. [52] showed opposing results and observed increased abundance of *Enterobacteriaceae* in patients undergoing colon resection who received a probiotic. The explanation of this phenomenon is not clear. One reason might be too short of a probiotic administration to reduce potential pathogen counts, while another could be associated with oatmeal used as a prebiotic, which could act as a substrate for intestinal bacteria, and the third one is that lactobacilli given orally did not survive the passage through the gastrointestinal tract. Another explanation is a different response of *Enterobacteriaceae* genera to probiotic administration (reduction in the numbers of one genera by the probiotic may result in an expansion of another). This is also of interest as lipopolysaccharide (LPS) attached to the membrane surface of Gram-negative microbes [77,78] may result in enhanced virulence phenotype expression [26]. In severe injuries, more virulent pathogens may predominate in the intestinal ecosystem [27], disrupt the intestinal barrier structure, and function and facilitate bacterial translocation resulting in SSIs and SRCs. 

The steady state composition of gut microbiota is crucial in maintaining gut homeostasis [79]. The mechanisms that are implicated in the pathogenesis of complications in patients in the perioperative period are complex. Initially, a healthy microbiota produces lactic acid, which is metabolized to short chain fatty acids (SCFAs), the latter ones are directly related to fecal *Bifidobacterium* count [66]. SCFAs, predominantly butyrate, are crucial for proper gut barrier structure and function [80,81]. After abdominal surgeries and in the course of multiple nonsurgical diseases, beneficial butyrate, acetate, and propionate concentration diminish as a consequence of the deterioration of lactic acid metabolism, as well as fasting [82]. Butyrate, apart from being an energy source for colonocytes, stimulates mucus production and tight junction proteins synthesis [75]. It has been found to inhibit the expression of virulence genes [83] and restrict the growth of *Pseudomonas aueroginosa*, a collagenase producer, implicated in the pathogenesis of anastomotic leakage [84,85]. Butyrate controls the function of regulatory T cells in a microbe-associated context [86] and suppresses inflammation via nuclear factor kappa-light-chain-enhancer of activated B cells (NF-kB) signaling [87]. It also stabilizes the hypoxia inducible factor involved in the augmentation of the barrier function [88]. This meta-analysis shows that the concentrations of acetic, butyric, and propionic acids were elevated in patients supplemented with probiotics. Surprisingly, a meta-regression indicated that the longer duration of probiotic intervention, the smaller the effect size for propionic acid. This seems to be in contrast with mechanistic studies in which propionic acid was discovered to act as an immunosuppressant [89]. This metabolite possesses anti-fungal and anti-bacterial effects [90] responsible for the inhibition of invasion genes in *Salmonella typhimurium.* Propionic acid is able to diminish the synthesis of eicosanoids via lowering the activity of cyclooxygenase [91,92]. Although the acid may inhibit mitogen-activating lymphocytes proliferation, different studies found that the inhibitory effects may be positively correlated with its concentration [93,94,95]. The discrepancies between concentrations inside and outside the visceral compartment may at least partly explain the observed results. It should be pointed out that this data was extracted from four studies, so the results need to be interpreted with caution [40,44,55,67]. More studies evaluating SCFAs concentration in surgical patients are needed to confirm this finding.

It was also found that in patients supplemented with pro-/synbiotics, the concentration of CRP and IL-6 were significantly decreased in comparison to non-treated patients. As antigens flow through the disrupted intestinal barrier, the activation of the immune response in *lamina propria* and the production of inflammatory mediators take place. IL-6 and CRP were found to be at higher serum concentrations in patients with low DAO activity following the surgery [58]. This is crucial as DAO being produced at the tip of the villi reflects the integrity of the small intestine barrier. The enzyme serum concentration is of small bowel origin [96,97,98] and its activity was found to be diminished following major hepatectomy [40,58,67]. This study shows that pro-/synbiotic intervention significantly lowered the concentration of IL-6 and CRP. The body of evidence states that IL-6 signaling plays a pivotal role in epithelial stem cells and intraepithelial lymphocytes proliferation and may be involved in wound healing [99]. Recently, Kuhn et al. [100] discovered that intraepithelial lymphocyte-derived IL-6 served positively toward barrier function via claudin-1 protein expression and increased mucus thickness [100]. Although CRP production in hepatocytes was found not to be influenced by medical therapies [101], the most recent meta-analysis by Mazidi et al. proved that probiotic administration may significantly reduce serum CRP with a weighted mean difference (WMD) of −1.35 mg/L; however, that study was not limited to surgical patients only [102]. 

Gut-derived bacteremia is a result of elevated intestinal permeability which further makes antigens flow through the epithelium, elevate serum inflammatory mediators [58], and enhance bacterial translocation to mesenteric lymph nodes after interventions such as a hepatectomy [103] and an esophagectomy [104]. In this study, it was not possible to demonstrate that microbial intervention diminished the risk of bacterial translocation. However, studies evaluating the bacterial translocation were based on culture-based methods and such methodology was valid to evaluate the presence of well-cultured bacteria only [66]. Culture-independent molecular techniques and sophisticated bioinformatic analyses should therefore be implemented in future trials to evaluate bacterial translocation and assess the functionality of translocated microorganisms in patients in perioperative periods.

This updated systematic review found that patients treated perioperatively with pro-/synbiotics had lower relative risk toward (i) abdominal distention, (ii) diarrhea, (iii) pneumonia, (iv) sepsis, (v) superficial incisional infection, (vi) urinary tract infection, (vii) duration of antibiotic therapy, (viii) duration of postoperative pyrexia, (ix) time of fluid introduction and (x) solid diet, and (xi) duration of hospital stay, and supports other observations [28,29,32].

This study also shows that biochemical parameters associated with the gut barrier were improved in patients treated with pro-/synbiotics, supporting the hypothesis that SSIs and SRCs are actually in large part sourced from the patient’s own gut flora. This is in line with a recent SR by Lederer et al. [105] who reported that the gut microbiome was responsible for postoperative complications including anastomotic leakage and wound infection. The data was not robust enough to establish recommendations for the use of beneficial bacteria in SSIs/SRCs prevention. The limitations of the available data did not allow us to determine which probiotics strain is the optimal choice, particular clinical situations where they could prove beneficial, how long the intervention should last, and the optimal dose of the supplement. The study was unable to establish that synbiotics should be used first-line to reduce specific SSIs and SRCs, which contrasts with the network meta-analysis by Kasatpibal et al. [28]. Apart from different methodological approach, this study included more patients (2952 vs. 3028) but excluded studies in a non-English language that may partly explain the discrepancies. Therefore, on the basis of the results of this study, microbial supplements in general, without strain recommendation in perioperative period, could be advocated. Taking into account the documented stability and safety of probiotics available on the market, the findings could explain the lack of current implementation of probiotics/synbiotics into SSIs/SRCs prevention clinical guidelines. More high-quality studies are needed to draw detailed protocols to evaluate particular probiotic strains, optimal duration of their supplementation, objective outcomes measurements, and maybe even stratify by surgery types to understand the roles. Nevertheless, the evidence is strong to already support dietary supplementation with probiotics in patients undergoing major abdominal surgeries. This topic seems to be of high priority as Berrios-Torres et al. [4] in their recent Centers for Disease Control and Prevention Guideline for the Prevention of Surgical Site Infection stated that antimicrobial prophylaxis should be administered only when indicated based on published clinical practice guidelines. The evidence is mounting that the longer post-surgical antibiotic administration, the greater the frequency of SSIs [1]. Antibiotic administration was found to elevate the risk toward inflammatory disorders, predominantly due to commensal bacteria translocation through the gut barrier, thus disturbing the microecological niche within the gut [106]. Also, antibiotic gut decontamination may activate dormant spores, which consequently results in severe infectious complications [107]. Recently, the 6th National Audit Project of the Royal College of Anaesthetists reported antibiotic-induced life-threatening anaphylaxis as well [108]. However, one of the current widely agreed and recommended intervention to decrease the incidence of SSIs/SRCs is perioperative antibiotic administration. 

Postsurgical complications (PSCs) are currently one of the most challenging health care issues worldwide [1,2]. Moreover, these unpredictable post-surgical events result in unscheduled readmissions, extended antibiotic therapy, and elevated mortality rate, but importantly generate additional costs of treatment. For example, Tanner et al., evaluated that in the U.K., SSIs secondary to colorectal surgery generated an extra cost of more than £10.000 with only 15% met in primary care [109]. More recently, Straatman et al. [110] pointed that in Netherlands, complications following major abdominal surgery may generate as much as 240% higher costs of treatment, depending on the clinical course of PSC. In the USA, the mean cost for a hospital stay was found to be approximately twice as high in patients with complications compared with those suffering from no PSCs. Consequently, total profit margin was estimated to be about 5.7% lower in patients with complications [111]. On the other hand, as reported by Keenan et al. [112], introducing a preventive strategy, e.g., SSI bundle in colorectal surgery, may significantly diminish the incidence of SSIs, and consequently, health care costs. As our paper provides evidence linking PSCs to host intestinal microenvironment, maintaining healthy microbiota—at least during the hospital stay—to reducing the incidence of these life-threatening events seems to be one of these cost-effective regimens [6,7,8]. Indeed, our study has shown that probiotic intervention significantly decreased the duration of antibiotic therapy (SMD: −0.597, 95% CI: −1.093, −0.10, *p* = 0.018) and overall length of hospital stay (SMD: −0.479, 95% CI: (−0.660, −0.297, *p* = 0.0000002). The reduction of these variables, together with the lowest incidence of PSCs reported in our study, extrapolate to a reduction in the cost of a patient’s stay in a hospital. This is in line with the assumptions made recently by Wu et al. [34] who analyzed two studies of Liu et al. [50,51] and reported a lower hospital charge concerning patients receiving probiotics in comparison to the placebo groups. Finally, it was concluded [34] that probiotic prophylaxis in surgery wards may decrease the hospital costs. 

Several limitations of this MA require underlining. These include (i) a small number of double-blind clinical trials; (ii) heterogeneous study aims, patient groups, intervention characteristics, and study targets; (iii) a limited number of reported outcomes; and (iv) meta-regression analyses were conducted only for exploratory reasons due to different subsets of patients and treatments. The overall moderate quality of the studies may have significantly influenced the study outcomes. Nevertheless, despite these limitations, this is the first, comprehensive SR/MA that shows a beneficial effect of pro-/synbiotics in reducing the incidence of SSIs/SRCs likely via modulating gut related immune response and production of SCFA.

In conclusion, our MA supports that pro-/synbiotics as a class can have an effect on the outcome, but more granular data on particular types and concentrations cannot be recommended. The effect on SSIs/SRCs is complex, including the modulation of CRP and WBC counts, as well as alteration of SCFAs synthesis and others that need further clarification. More high-quality studies are needed to draw detailed protocols to evaluate particular probiotic strains and optimal duration of their supplementation in patients undergoing surgical procedures. However, the evidence presented in this systematic review strongly supports that dietary supplementation with probiotics in patients undergoing major abdominal surgeries has a beneficial effect.

## Figures and Tables

**Table 1:** Study characteristics.

Study	Reference	Study (Country)	Study Description					Treatment Description				Subjects Description			
			Study Focus/Primary Study Outcome	Blinding	Trial Duration (Days)	ROB*	Operation Name	Duration of Probiotic Therapy Pre/Postoperatively (Days)	Probiotic/Synbiotic Content	Probiotic Dose	Comparator	*N* Total Randomized/Analysed	Age (Years)	Male (%)	Primary Disease
1	[41]	Aisu 2015 (Japan)	SSIs and the immune response, intestinal microbiota, and surgical outcome	Psr	ND	2	CRC surgery	3–15/NR	*Enterococcus faecalis* T110, *Clostridium butyricum* TO‑A, *Bacillus mesentericus* TO‑A	2 mg, 10 mg, 10 mg; 6 tablets/day	No intervention	156/156	68.57 ± 12.49	91 (58.33)	CRC
2	[45]	Anderson 2003 (U.K.)	BT, gastric colonisation, systemic inflammation, and septic morbidity	DB	12	5	Elective laparotomy	12/4	*Lactobacillus acidophilus* La5, *Lactobacillus bulgaricus, Bifidobacterium lactis* Bb-12, *Streptococcus thermophilus*; Prebiotic: oligofructose	4 × 10^9^ CFU; 16 g; 3 × day	PBO	137/137	71 ^#^	80 (58.39)	GI malignancy
3	[46]	Diepenhorst 2011 (The Netherlands)	BT, intestinal barrier function	DB	14	3	Elective pylorus-preserving pancreaticoduodenectomy	7/7	*Lactobacillus acidophilus* W70, *Lactobacillus casei* W56, *Lactobacillus salivarius* W24, *Lactococcus lactis* W58, *Bifidobacterium* *Bifidum* W23, *Bifidobacterium infanti*s W52	3 g; 2 × day (an equivalent of 10^10^ CFU)	Standard care	20/20	64 ^#^	10 (50)	Periampullary or ampullary pancreatic malignancy
									*Lactobacillus acidophilus* W70, *Lactobacillus casei* W56, *Lactobacillus salivarius* W24, *Lactococcus lactis* W58, *Bifidobacterium* *Bifidum* W23, *Bifidobacterium infanti*s W52 + SDD				60 ^#^	9 (45)	
4	[43]	Eguchi 2011 (Japan)	Infectious complications	OL	16	1	Living donor LT	2/14	*Lactobacillus casei* Strain Shirota, *Bifidobacterium breve* Strain Yakult; Prebiotic: GOS	20 mg + 15 mg + 15 mg/3 × day	PBO	50/50	56.5 ± NR	29 (58)	Liver cirrhosis due to HCV
5	[47]	Flesch 2017 (Brazil)	Surgical wound infection	DB	19	2	Colorectal resection	5/14	*Lactobacillus acidophilus* NCFM, *Lactobacillus rhamnosus* HN001, *Lactobacillus paracasei Lactobacillus plantarum* c-37, *Bifidobacterium lactis* HN019; Prebiotic: FOS	10^9^ each, 6 g/2 sachets 2 × day	PBO	100/91	62.93 ± 12.32	37 (40.66)	Colorectal adenocarcinoma
6	[64]	Grąt 2017 (Poland)	Pre- and post-transplant patient outcomes	DB	Varia, depending on the listing for LT	6	LT	Varia depending on listing for LT, up to 10 weeks	*Lactococcus lactis* PB411, *Lactobacillus casei* PB121, *Lactobacillus acidophilus* PB111, *Bifidobacterium bifidum* PB211	3 × 10^9^ CFU	PBO	55/44	50.95	34 (77.27)	ALD
7	[48]	Horvat 2010 (Slovenia)	Systemic inflammatory response and clinical outcome	DB	NR	3	Abdominal surgery	3/NR	*Pediacoccus pentosaceus* 5-33:3, *Leuconostoc mesenteroides* 32–77:1, *Lactobacillus paracasei* subsp. *Paracasei* 19, *Lactobacillus plantarum* 2362; Prebiotic: 2.5 g betaglucan, 2.5 g inulin, 2.5 g pectin, 2.5 g resistant starch	40 billion, 10 g of fibers, 2 × day	Bowel cleansing	76/40	62 ^#^	20 (50)	Colon adenocarcinoma
											Prebiotic	76/48	63.25 ^#^	21 (44)	
8	[40]	Kanazawa 2005 (Japan)	Intestinal integrity, microflora, and surgical outcome	NR	14	1	Combined liver and extrahepatic bile duct resection with hepaticojejunostomy	0/14	*Bifidobacterium breve* Strain Yakult, *Lactobacillus casei* Strain Shirota; Prebiotic: GOS **	10^8^/g each; 3 g day; 12 g/day	No intervention	54/44	63.75 ± 9.64	29 (65.91)	Perihilar cholangiocarcinoma
9	[44]	Komatsu 2016 (Japan)	Surgical outcome	OL	≤17	5	Laparoscopy	7–11/6	*Lactobacillus casei* strain Strain Shirota; Prevbiotic: GOS, *Bifidobacterium breve* Strain Yakult.	4 × 10^10^, 2.5 g, 1 × 10^10^	No intervention	370/362	67.23 ± 11.11	210 (58.01)	Elective laparoscopic colorectal surgery
10	[49]	Kotzampassi 2015 (Greece)	Prophylaxis for complications after colorectal surgery	DB	16	5	Colorectal surgery for cancer.	1/14	*Lacctobacillus acidophilus* LA-5, *Lactobacillus plantarum*, *Bifidobacterium lactis* BB-12, *Saccharomyces boulardii*	1.75 × 10^9^ CFU, 0.5 × 10^9^ CFU, 1.75 × 10^9^, 1.5 × 10^9^ CFU per capsule, 2 × day	PBO	168/164	66.14 ± 11.69	115 (70.12)	CRC
11	[42]	Liu 2010 (China)	Gut barrier function and the surgical outcome	DB	16	4	Laparotomy	6/10	*Lactobacillus plantarum* CGMCC No. 1258, *Lactobacillus acidophilus* LA-11, *Bifidobacterium longum* BL-88	2.6 × 10^14^ CFU, 2 g/day	PBO	114/100	65.5 ± 10.45	59 (59)	CRC
12	[50]	Liu 2013 (China)	Serum zonulin concentrations and postoperative infectious complications	DB	16	5	Colorectsal carcinoma surgery	6/10	*Lactobacillus plantarum* CGMCC No. 1258, *Lactobacillus acidophilus* LA-11, *Bifidobacterium longum* BL-88	2.6 × 10^14^ CFU, 2 g/day	PBO	161/150	65.06 ± 11.73	78 (52)	CRC
13	[51]	Liu 2015 (China)	Serum zonulin levels and postoperative infectious complications	DB	16	5	Colectomy + resection for metastatic tumor/segmental hepatectomy	6/10	*Lactobacillus plantarum* CGMCC No. 1258, *Lactobacillus acidophilus* LA-11, *Bifidobacterium longum* BL-88	2.6 × 10^14^ CFU, 2 g/day	PBO	134/117	62.84 ± 17.17	70 (59.83)	Colon cancer + Colorectal liver metastases
14	[52]	Mangell 2012 (Sweden)	Intestinal load of potentially pathogenic bacteria, BT, and cell proliferation	DB	13	4	Colonic resection	8/5	*Lactobacillus plantarum* 299v	10^11^ CFU	PBO	72/64	72 ^#^	36 (56.25)	Adenocarcinoma
15	[53]	Mcnaught 2002 (U.K.)	BT, gastric colonization, and septic complications	OL	9	1	Major abdominal surgery	7–12/4–9	*Lactobacillus plantarum* 299v	10^7^/mL; preoperatively 4000 mL, postoperatively 800 mL	No intervention	129/129	68.5 ^#^	75 (58.14)	CRC
16	[65]	Mizuta 2016 (Japan)	Immune functions, systemic inflammatory responses, postoperative infectious complications	SB	≤28	2	CRC resection	7–14/7	*Bifidobacterium Longum* BB536	5 × 10^10^ CFU, 2 g	No intervention	60/60	70.01 ± 9.96	35 (58.33)	CRC
17	[54]	Nomura 2007 (Japan)	Surgical outcome	NR	≥3	1	Pancreaticoduodenectomy, Whipple	3–15/until discharge	*Enterococcus faeceali*s T-110, *Clostridium butyricum* TO-A, *Bacillus mesentericus* TO-A	6 × 10^7^ CFU	No intervention	70/64	66 ^#^	39 (60.94)	Pancreatico-billiarty disease
18	[55]	Okazaki 2013 (Japan)	Gut microbiota, infectious complications	OL	17	1	Abdominal surgery	7/10	*Lactobacillus casei* Strain Shirota and BBG-01, *Bifidobacterium breve* Strain Yakult; Prebiotic: GOS	Biolactis powder (1 g/day) and BBG-01 (1 g/day), GOS: 5 g, 3 × day	No intervention	53/48	78.5 ^#^	26 (54.17)	Upper digestive illness
19	[63]	Rammohan 2015 (India)	Postoperative infectious complications, clinical outcome	SB (patients)	15	3	Frey procedure for chronic hepatitis	5/10	*Streptococcus faecalis* T-110, *Clostridium butyricum* TOA, *Bacillus mesentericus* TO-A, *Lactobacillus sporogenes*; Prebiotic: FOS	60 million, 4 million, 2 million, 100 million,	PBO	79/75	43.29 ± 8.96	48 (64)	Chronic hepatitis
20	[72]	Rayes 2007 (Germany/U.K.)	Postoperative bacterial infection	DB	9	2	Pylorus-preserving Pancreatoduodenectomy	1/8	*Pediacoccus pentosaceus* 5–33:3; *Leuconostoc mesenteroides* 77:1; *Lactobacillus paracasei* subspecies *paracasei* F19; *Lactobacillus plantarum* 2362; Prebiotic: bioactive fibers—2.5 g of each betaglucan, inulin, pectin, and resistant starch,	10^10^, 10 g	Fiber	89/80	58.5 ± NR	45 (56.3)	Carcinoma (pancreas)
21	[71]	Rayes 2005 (Germany/U.K.)	Infectious complications	DB	14	3	LT	0/14	*Pediacoccus pentosaceus* 5–33:3; *Leuconostoc mesenteroides* 77:1; *Lactobacillus paracasei* subspecies *paracasei* F19; *Lactobacillus plantarum* 2362; Prebiotic: bioactive fibers—2.5 g of each betaglucan, inulin, pectin, and resistant starch	10^10^, 20 g	Fiber	66/66	51.5 ± 2	38 (57.6)	Na
22	[70]	Rayes 2002 a (Multicenter)	Early postoperative infections	OL	12	0	LT	0/12	*Lactobacillus plantarum* 299v; 2 × day	1 × 10^9^, oat fibers	PBO + fiber	105/69	48.47 ± 2.49	30 (47.6)	Na
23	[69]	Rayes 2002 (Germany)	Postoperative bacterial infection, clinical outcome	OL	4	0	Major abdominal surgery	0/4	*Lactobacillus plantarum* 299; Prebiotic: oat fiber	1 × 10^9^	PBO + fiber	90/60	60.5 ± 13.59	30 (50)	Liver, pancreatic, gastric resection
24	[73]	Rayes 2012 (Germany)	Liver regeneration after hepatectomy	DB	11	2	Hepatectomy	1/10	*Pediacoccus pentosaceus* 5–33:3; *Leuconostoc mesenteroides* 77:1; *Lactobacillus paracasei* subspecies *paracasei* F19; *Lactobacillus plantarum* 2362; Prebiotic: bioactive fibers—2.5 g of each betaglucan, inulin, pectin, and resistant starch	10^10^, 20 g	Fiber	19/19	60.05 ± 13.89	14 (73.7)	Colorectal metastasis
25	[62]	Reddy 2007 (Denmark/U.K.)	Prevalence of Enterobacteriaceae, inflammatory response including septic morbidity	OL		1	Elective CRC surgery	1/0	*Lactobacillus acidophilus* La5, *Lactobacillus bulgaricus*, *Bifidobacterium lactis*, BB-12, *Streptococcus thermophilus*; Prebiotic: oligorfructose	4 × 10^9^ CFU, 15 g, 2 × day	Neomycin + MBP	88/42	70.6 ^#^	22 (52.4)	Anterior resection
26	[61]	Sadahiro 2014 (Japan)	Incisional SSI, organ/space SSI, remote infection, leakage, CD toxin	DB	18	6	Curative resection of CRC	7/11	*Bifidobacterium bifidum*; Prebiotic: multooligossacharide	1 × 10^9^/day	Antibiotic, mechanical bowel preparation	294/194	66.7 ± 10.72	107 (55.2)	CRC
27	[60]	Sommacal 2015 (Brazil)	Postoperative morbidity and mortality	DB	14	7	Periampullary cancer: resective and palliative surgery	4/10	*Lactobacillus acidophilus* 10, *Lactobacillus rhamnosus* HS 111, *Lactobacillus casei* 10, *Bifidobacterium bifidum*; Prebiotic: FOS	1 × 10^9^ CFU, 1 × 10^9^ CFU, 1 × 10^9^ CFU, 1 × 10^9^ CFU, 100 mg	PBO	48/46	59.5 ^#^	NR	Periampullary cancer
28	[67]	Sugawara 2006 (Japan)	Intestinal barrier function, immune responses, systemic inflammatory responses, microflora, and surgical outcome	OL	28	2	Liver and extrahepatic bile duct resection with hepaticojejunostomy	14/14	*Lactobacillus casei* strain Shirota, *Bifidobacterium breve* strain Yakult; Prebiotic: GOS	80 mL: 4 × 10^10^; 100 mL: 1 × 10^10^; 15 g/day	Synbiotic only post-operatively	101/81	63.15 ± 8.84	46 (56.79)	Perihilar cholangiocarcinoma
29	[59]	Tanaka 2012 (Japan)	Postoperative infections	SB	21	3	Oesophagectomy	1/21	*Lactobacillus casei* strain Shirota, *Bifidobacterium breve* strain Yakult; Prebiotic: GOS	1 × 10^10^/g, 1 × 10^10^/g; (PRE:3 g/day; POST: 2 g/day) GOS (PRE:15 g, POST:10 g)	*Streptococcus faecalis*	64/64	62.15 ± 7.74	51 (79.7)	Oesophagal cancer
30	[58]	Usami 2011 (Japan)	Intestinal integrity, systemic inflammatory response, and microflora, surgical outcome	OL	26	4	Hepatic surgery	14/12	*Lactobacillus casei* strain Shirota, *Bifidobacterium breve* strain Yakult; Prebiotic: GOS	1 × 10^8^/g, 1 × 10^8^/g; 10 g	No intervention	67/61	65.42 ± 9.86	55 (90.2)	Primary or metastatic liver cancer
31	[39]	Yang 2016 (China)	Postoperative infections	DB	12	5	Radical CRC resection	5/7	*Bifidobacterium longum, Lactobacillus acidophilus Enterococcus faecalis*	≥1.0 × 10^7^ CFU/g, ≥1.0 × 10^7^ CFU/g, ≥1.0 × 10^7^ CFU/g)	PBO	79/60	63.03 ± 11.70	27 (45)	CRC
32	[57]	Yokoyama 2014 (Japan)	Intestinal microenvironment, BT to mlns, postoperative bacteraemia	OL	21	5	Oesophagectomy	7/14	PRE:*Lactobacillus casei* strain Strain Shirota, *Bifidobacterium breve* strain Strain Yakult; Prebiotic: 15 g GOS; POST:*Lactobacillus casei* strain Strain Shirota *Bifidobacterium breve* strain Strain Yakult; Prebiotic: 15 g GOS	PRE: 4 × 10^10^, 1 × 10^10^, 15 g; POST: 1 × 10^8^/g; 1 × 10^8^/g; 15 g	No intervention	42/42	65.5 ^#^	37 (88.1)	Oesophagal cancer
33	[66]	Yokoyama 2016 (Japan)	BT to mlns and blood, postoperative infectious complications	OL	7	2	Pancreatoduodenectomy	7/0	Lactobacillus casei Strain Shirota, Bifidobacterium breve strain Strain Yakult; Prebiotic: GOS	80 mL: 4 × 10^10^; 100 mL: 1 × 10^10^; 15 g/day	No intervention	45/44	65 ^#^	12 (27.27)	Pancreatic cancer
34	[56]	Zhang 2012 (China)	Postoperative infections and related complications	DB	3	5	Radical CRC resection with laparotomy	3/0	*Bifidobacterium longum, Lactobacillus acidophilus, Enterococcus faecalis*	0.21 g (10^8^ CFU/g)	PBO	60/60	64.5 ^#^	24 (60)	CRC
35	[68]	Zhang 2013 (Australia)	Assessing the impact on bacterial sepsis and wound complications	OL	?	2	LT	0/?	*Lactobacillus Acidophilus* LA-14, *Lactobacillus Plantarum* 115, *Bifidobacterium Lactis* BL-04, *Lactobacillus Case*i LC-11, *Lactobacillus Rhamnosus* LR-32, *Lactobacillus Brevis* lbr-35; Prebiotic: fiber	15.5 × 10^9^; 5.0 × 10^9^; 2.0 × 10^9^; 1.5 × 10^9^; 1.5 × 10^9^; 1.5 × 10^9^ CFU	Fiber	67/67	56.01 ± 10.98	36 (53.73)	NR

*—number of low risk judgements; **—enetral feeding, ^#^—median, CFU—colony forming units, DB—double blind, SB—single blind, CRC—colorectal cancer, GI—gastrointestinal, LT—liver transplantation, GOS—galactoologosaccharides, FOS—fructooligosaccharides, OL—open label, PsR—pseudorandomisation, SDD—standard decontamination of the digestive tract, BT—bacterial transolcation, MLN—mesenteric lymph node, ALD—alcoholic liver disease, CRC—colorectal cancer, SDD—selective decontamination of the digestive tract.

**Table 2:** Gut microbiota alterations following probiotic treatment.

Reference	Country	Gut Microbiota Changes after the Surgery/Intervention
Aisu 2015	Japan	Probiotic group: the mean proportion of *Bifidobacterium* increased between 4.6 ± 1.97 and 9.1 ± 1.89%. No-probiotic group: the mean proportion of *Bifidobacterium* decreased between 7.06(1.95)% And 5.53(±1.93)
Eguchi 2011	Japan	No significant changes in bacterial species abundance between the groups. In 25% of patients under immunosuppression *Enterococcus* spp evident in both groups
Grąt 2017	Poland	Probiotic group:*Bacteroides* spp. count increased in comparison to pre-trial values (*p* = 0.008). *Enterococcus* spp. abundance significantly increased (*p* = 0.04) and a tendency towards increased number of *Lactobacillus* spp. (*p* = 0.07) as compared to no-probiotic group
Kanazawa 2005	Japan	Synbiotic group: beneficial bacteria (including *Lactobacillus* and *Bifidobacterium*) count increased after surgery, in comparison to controls (*p* < 0.05). No-synbiotic group: harmful microorganisms (including *Enterobacteriaceae, Pseudomonas*, and *Candida*) increased in comparison to synbiotic group (*p* < 0.05). *Enterococci* abundance increased after surgery in both groups, with no significant intergroup differences.
Komatsu 2016	Japan	Synbiotic group: Total bacteria, dominant obligate anaerobes (such as *Clostridium leptum* subgroup or *Bifidobacterium*), and facultative anaerobes (*Lactobacillus* species) significantly increased. The abundance of *Enterobacteriaceae*, *Staphylococcus* (MSCNS), and *Pseudomonas* decreased compared to the control group (*p* < 0.05). *Bifidobacterium* and *L. casei* subgroup numbers and *C. perfringens*, *L. gasseri* subgroup, *L. reuteri* subgroup, *L. ruminis* subgroup, and *L. sakei* subgroup increased and decreased respectively regarding preoperative concentrations (*p* < 0.05). No synbiotic group: total bacteria, dominant obligate anaerobes (*C. coccoides* group, *C. leptum* subgroup, *Bacteroides fragilis* group, *Bifidobacterium, Prevotella*, and *Lactobacillus* species) counts decreased while the numbers of *Enterobacteriaceae*, *Staphylococcus* (MSCNS), *Pseudomonas*, and *C. difficile* increased in comparison to the preoperative values (*p* < 0.05).
Liu 2010	China	Probiotic group:*Bifidobacterium* count increased in comparison to controls and preoperative values. *Enterobacteriaceae*, *Pseudomonas*, and *Candida* numbers were decreased compared to placebo group (*p* < 0.05). Probiotic bacterial richness was enhanced when compared to healthy volunteers and the control group (*p* < 0.05). A higher similarity to the healthy volunteers compared with the control group (*p* < 0.05). No probiotic group: *Enterobacteriaceae, Pseudomonas* and *Candida* numbers increased compared to probiotic group (*p* < 0.05) *Enterococci* abundance increased in both groups.
Mangell 2012	Sweden	Probiotic group:*Enterobacteriaceae* count increased significantly in comparison to placebo (*p* < 0.001) but not regarding preoperatively values.
Mizuta 2016	Japan	Probiotic group:*Firmicutes* decreased (62.31% vs. 56.51%) and *Actinobacteria* increased (0.7% vs. 1.71%) in comparison to control group (*p* < 0.05). No-probiotic group: *Bacteroidetes* (24.52% vs. 32.8%) and *Proteobacteria* (1.74% vs. 3.54%) numbers increased and *Firmicutes* (66.57% vs. 56.82%) and unclassified bacterial groups (0.5% vs. 0.37%) abundance decreased compared to before the surgery period.
Okazaki 2013	Japan	Synbiotic group: Before surgery *Bifidobacteria* count and numbers of *Enterobacteriaceae* and *Pseudomonas* were significantly increased and decreased, respectively, in comparison to the pre-trial values and the control group (*p* < 0.05). *Bifidobacterium* abundance was significantly increased while *Enterobacteriaceae* and *Staphylococcus* bacteria counts decreased postoperatively in comparison to controls. No-synbiotic group: *Bifidobacterium* number gradually decreased
Sugawara 2006	Japan	Pre-and post-operative probiotic group: *Bifidobacterium* number increased significantly after preoperative treatment (*p* < 0.05), as well as *Lactobacillus* but with no statistical difference (*p* > 0.05). *Bifidobacterium* abundance 1 day before hepatectomy was higher and lower for *Candida* in comparison to the only pre-surgery probiotic group. Anaerobic bacteria numbers were unchanged before and after surgery between the two groups, without intergroup differences.
Tanaka 2012	Japan	Synbiotic group: *Bifidobacterium* and total *Lactobacillus* numbers were significantly higher (*p* < 0.01) when compared to controls. Postoperatively (day 7) the abundance of *Clostridium coccoides* group (*p* < 0.01); *C. leptum* subgroup (*p* < 0.01); *Bacteroides fragilis* group (*p* < 0.05); *Bifidobacterium* (*p* < 0.01); *Atopobium* cluster (*p* < 0.05), *Prevotella* (*p* < 0.01), and *Lactobacillus* (*p* < 0.01) significantly decreased when compared to the pre-operative time point. *Bifidobacterium* and *Lactobacillus* species count were not decreased, but were higher when compared to controls. *Enterobacteriaceae, Staphylococcus*, and *Pseudomonas* species numbers were significantly lower in comparison to the second group patients. Collectively (3 weeks post-surgery) *Bifidobacterium* abundance was significantly higher and *Enterobacteriaceae* count was lower in the synbiotic group (*p* < 0.05).
Usami 2011	Japan	Synbiotic group: Fecal anaerobic bacteria, including *Bacteroidaceae*, as well as *Bifidobacterium* genus were decreased compared to before the trial (post-operative days 6–8). The numbers of *Candida* were increased in this time point. In contrast, two weeks after the surgery, these numbers started to resemble values before hepatectomy (*Bacteroidaceae*: 10.0 ± 0.4 vs. 10.1 ± 0.3, *Bifidobacterium*: 10.0 ± 0.7 vs. 10.0 ± 0.6, *Candida*: 3.4 ± 1.4 vs. 3.1 ± 1.0 log10 CFU/g of feces. No-synbiotic group: Two weeks after the surgery, particular bacteria numbers started to resemble values before hepatectomy (*Bacteroidaceae*: 10.0 ± 0.5 vs. 9.9 ± 0.4, *Bifidobacterium*: 9.8 ± 0.8 vs. 9.5 ± 0.7, *Candida*: 4.1 ± 1.6 vs. 4.1 ± 1.9 log10 CFU/g of feces. Subgroup comparison between normal liver and chronic liver damage, including chronic hepatitis, liver fibrosis, and cirrhosis in either group found no significant differences
Yokoyama 2014	Japan	Synbiotic group: A week post-surgery, *Bifidobacterium* and *Lactobacillus* counts increased and *Enterobacteriaceae* and *Pseudomonas* decreased in comparison to pre-operative values and the control group (*p* < 0.05). The numbers of *Staphylococus*, *Pseudomonas*, and *Enterobacteriaceae* were significantly decreased 21 days post-surgery when compared to the no-synbiotic group and pre-surgery time (except for *Pseudomonas*) No-synbiotic group: *Pseudomonas, Staphylococcus*, and *Enterobacteriaceae* levels were increased post-operatively in comparison to the intervention group (*p* < 0.05).
Zhang 2012	China	Probiotic group: During preoperative treatment (3 days before surgery), the reversal of the *Bifidobacterium/E. coli* ratio inversion in comparison to day–6 (0.26 ± 0.32 and 1.26 ± 0.28 log10/g, respectively, *p* < 0.001) and controls (1.26 ± 0.28 and 0.27 ± 0.34 log10/g, respectively, *p* < 0.001). Postoperatively decreased *E coli* count compared to controls (8.29 ± 0.27 log10/g and 9.67 ± 0.17 log10/g, respectively, *p* < 0.001), and *B. longum* increased (8.43 ± 0.17 log10/g and 7.94 ± 0.11 log10/g, respectively; *p* < 0.001). No-probiotic group: Postoperative *Bifidobacterium/E. coli* ratio inversion in comparison to 6 days before surgery (0.14 ± 0.20 and 0.26 ± 0.32, respectively, *p* < 0.001) and probiotic group (0.14 ± 0.20 and 1.73 ± 0.22, *p* < 0.001).

**Table 3:** Primary outcomes associated with gut barrier implicated in potential mechanisms of probiotic/synbiotic action.

Outcome	SMD (95% CI)	Z-Value	References	Heterogeneity	Tau	Intercept (95% CI) ^†^	Meta-Regression Coefficients
CRP	−0.40 (−0.79, −0.02)	−2.04 *p* = 0.041	Kanazawa, 2005 Yokoyama, 2014 Usami, 2011 Tanaka, 2012 Rayes, 2002 Sugawara, 2006	Q = 16.1 *p* = 0.007 (df = 5) I^2^ = 69	τ^2^ = 0.159 τ = 0.399	8.59 (−13.42, 30.59) *p* = 0.339	Dose: −0.32 (*p* = 0.158) Intervention: NOT ESTIMABLE Operation (Hepatobiliary vs. Gut): −0.69 (p = 0.075), (Mixed vs. Gut): −0.34, *p* = 0.515 ROB (Low vs. High): −0.28 (*p* = 0.539) Duration: −0.02 (*p* = 0.477) Timing (Post vs. Peri): 0.08 (*p* = 0.871)
IL-6	−0.41 (−0.70, −0.12)	−2.77 *p* = 0.006	Zhang, 2012 Usami, 2011 Sugawara, 2006 Mizuta, 2016	Q = 4.03 *p* = 0.258 (df = 3) I^2^ = 25.6	τ^2^ = 0.022 τ = 0.150	−2.18 (−39.73, 35.38) *p* = 0.826	Dose: −0.09 (*p* = 0.538) Intervention (Synbiotic vs. Probiotic): 0.36 (*p* = 0.159) Operation (Hepatobiliary vs. Gut): 0.36 (*p* = 0.159) ROB (Low vs. High): −0.27 (*p* = 0.383) Duration: 0.01 (*p* = 0.231) Timing (Pre vs. Peri): −0.22 (*p* = 0.580)
WBC	−0.60 (−1.45, 0.24)	−1.40 *p* = 0.162	Kanazawa, 2005 Yokoyama, 2014 Usami, 2011 Tanaka, 2012 Rayes, 2002a Sugawara, 2006	Q = 70 *p* < 0.0001 (df = 5) I^2^ = 93	τ^2^ = 1.033 τ = 1.016	0.09 (−38.14, 38.32) *p* = 0.995	Dose: −0.03 (*p* = 0.965) Intervention: NOT ESTIMABLE Operation (Mixed vs. Gut): −1.45 (*p* = 0.078) ROB (Low vs. High): −1.42 (*p* = 0.089) Duration: 0.05 (*p* = 0.515) Timing (Post vs. Peri): −1.13 (*p* = 0.223)
L/M	−0.28 (−0.82, 0.27)	−1.00 *p* = 0.316	Kanazawa, 2005 Liu, 2010 Liu, 2013 Sugawara, 2006	Q = 19.5 *p* = 0.0002 (df = 3) I^2^ = 85	τ^2^ = 0.257 τ = 0.507	8.66 (−14.75, 32.07) *p* = 0.252	Dose: −0.28 (*p* = 0.323) Intervention (Synbiotic vs. Probiotic): 0.46 (*p* = 0.435) Operation (Mixed vs. Gut): 0.46 (*p* = 0.435) ROB (Low vs. High): 0.46 (*p* = 0.435) Duration: −0.002 (*p* = 0.968) Timing (Post vs. Peri): 0.59 (*p* = 0.376)
Butyrate	0.67 (0.37, 0.97)	4.40 *p* = 0.00001	Kanazawa, 2005 Komatsu, 2016 Okazaki, 2013 Sugawara, 2006	Q = 5.04 *p* = 0.169 (df = 3) I^2^ = 40.4	τ^2^ = 0.037 τ = 0.193	1.37 (−8.79, 11.53) *p* = 0.622	Dose: NOT ESTIMABLE Intervention: NOT ESTIMABLE Operation: NOT ESTIMABLE ROB (Low vs. High): 0.22 (*p* = 0.572) Duration: 0.02 (*p* = 0.510) Timing (Post vs. Peri): 0.45 (*p* = 251)
Acetate	1.78 (0.80, 2.76)	3.55 *p* = 0.0004	Kanazawa, 2005 Komatsu, 2016 Okazaki, 2013 Sugawara, 2006	Q = 41.4 *p* < 0.0001 (df = 3) I^2^ = 93	τ^2^ = 0.912 τ = 0.955	2.65 (−26.40, 31.71) *p* = 0.732	Dose: NOT ESTIMABLE Intervention: NOT ESTIMABLE Operation: NOT ESTIMABLE ROB (Low vs. High): −0.27 (*p* = 0.851) Duration: −0.10 (*p* = 0.118) Timing (Post vs. Peri): −0.25 (*p* = 0.850)
Propionate	0.46 (0.18, 0.73)	3.23 *p* = 0.001	Kanazawa, 2005 Komatsu, 2016 Okazaki, 2013 Sugawara, 2006	Q = 4.58 *p* = 0.206 (df = 3) I^2^ = 34.4	τ^2^ = 0.028 τ = 0.166	−1.99 (−11.22, 7.24) *p* = 0.451	Dose: NOT ESTIMABLE Intervention: NOT ESTIMABLE Operation: NOT ESTIMABLE ROB (Low vs. High): −0.38 (*p* = 0.074) Duration: −0.04 (*p* = 0.049) Timing (Post vs. Peri): 0.18 (*p* = 0.675)

† Egger’s regression intercept test for asymmetry of the funnel plots; Dose – dose of probiotic (log), ROB – risk of bias, Post – post operation, Pre – pre operation, Peri – peri operation, SSI-surgical site infection.

## Supplementary Materials

The following are available online at http://www.mdpi.com/2077-0383/7/12/556/s1. Figure S1: The effect size (risk ratio) for the overall effects of probiotics in the prevention of pneumonia. Figure S2: The effect size (risk ratio) for the overall effects of probiotics in the prevention of surgical site infection. Table S1: The efficacy of probiotics to counteract surgery related complications (SRCs). Table S2: Risk of bias assessment.

## Data Source

Data presented in this manuscript were discussed in the presidential choice session of the WGO-GAT International Conference, Bangkok, 5–8 Dec 2018.
