# Supplementary material for: A Systematic Review, Meta-Analysis, and Meta-Regression Evaluating the Efficacy and Mechanisms of Action of Probiotics and Synbiotics in the Prevention of Surgical Site Infections and Surgery-Related Complications
Source: J Clin Med. 2018 Dec 16;7(12):556. doi: 10.3390/jcm7120556 (PMC6307089; doi:10.3390/jcm7120556)
Supplement: Supplementary file 1 [file jcm-07-00556-s001.pdf]

Pneumonia

Model	Study name	Risk ratio	Lower limit	Upper limit	Z-Value	p-Value
	Aisu, 2015	3.237	0.134	78.254	0.723	0.470
	Flesh, 2017	0.096	0.005	1.725	-1.591	0.112
	Kanazawa, 2005	0.364	0.016	8.468	-0.630	0.529
	Kotzampassi, 2015	0.212	0.047	0.950	-2.027	0.043
	Liu, 2010	0.400	0.081	1.966	-1.128	0.259
	Liu, 2013	0.300	0.086	1.047	-1.888	0.059
	Liu, 2015	0.773	0.283	2.107	-0.504	0.614
	Okazaki, 2013	0.920	0.141	6.007	-0.087	0.931
	Zhang, 2012	0.250	0.030	2.108	-1.274	0.203
	Yokoyama, 2014	1.400	0.528	3.713	0.676	0.499
	Yang, 2016	0.600	0.157	2.289	-0.748	0.455
	Tanaka, 2012	0.276	0.063	1.210	-1.707	0.088
	Reddy, 2007	0.550	0.054	5.612	-0.504	0.614
	Rayes, 2007	0.111	0.006	1.998	-1.490	0.136
	Rayes, 2005	0.333	0.014	7.897	-0.680	0.496
	Rayes, 2002a	0.258	0.031	2.182	-1.244	0.214
	Rayes, 2002	2.000	0.191	20.898	0.579	0.563
	Rayes, 2012	3.300	0.151	72.084	0.759	0.448
	Rammohan, 2015	0.615	0.109	3.474	-0.550	0.582
	Zhang, 2013	0.324	0.014	7.675	-0.698	0.485
	Sugawara, 2006	0.325	0.035	2.997	-0.991	0.322
	Yokoyama, 2016	1.000	0.067	15.000	0.000	1.000
Random		0.549	0.377	0.800	-3.120	0.002

Risk ratio and 95% CI

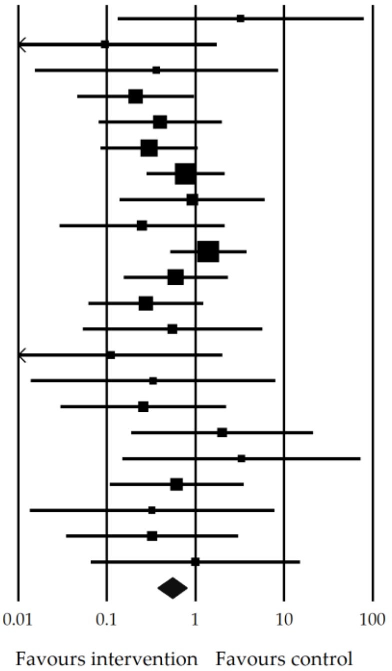

Figure S1. The effect size (risk ratio) for the overall effects of probiotics in the prevention of pneumonia.

SSI

Model	Study name	Risk ratio	Lower limit	Upper limit	Z-Value	p-Value
	Flesh, 2017	0.093	0.012	0.704	-2.300	0.021
	Kanazawa, 2005	0.548	0.156	1.918	-0.942	0.346
	Komatsu, 2016	0.761	0.500	1.159	-1.271	0.204
	Kotzampassi, 2015	0.357	0.147	0.867	-2.275	0.023
	Okazaki, 2013	0.184	0.023	1.460	-1.602	0.109
	Zhang, 2012	0.250	0.030	2.108	-1.274	0.203
	Zhang, 2013	0.388	0.081	1.863	-1.182	0.237
	Sugawara, 2006	0.325	0.070	1.517	-1.430	0.153
	Yokoyama, 2016	1.500	0.277	8.119	0.471	0.638
	Mizuta, 2016	0.655	0.288	1.491	-1.009	0.313
Random		0.553	0.392	0.781	-3.364	0.001

Risk ratio and 95% CI

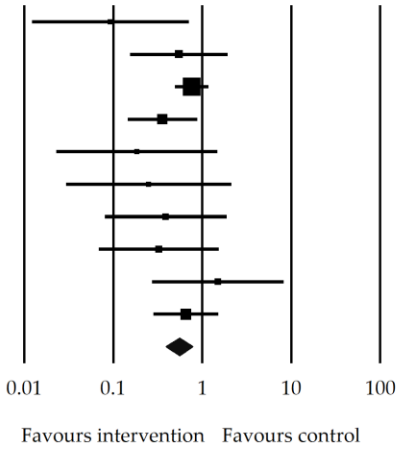

Figure S2. The effect size (risk ratio) for the overall effects of probiotics in the prevention of surgical site infection.

Table S1. The efficacy of probiotics to counteract surgery related complications (SRCs).

Outcome	RR (95%CI)	Z-value	References	Heterogeneity	Tau	Intercept (95%CI)†	Meta-regression coefficients
Abdominal distention	0.64<br>(0.47, 0.86)	-2.93<br>p=0.003	Liu, 2010<br>Liu, 2015<br>Yang, 2016<br>Zhang, 2013	Q=0.247<br>p=0.970 (df=3)<br>I^2=0	τ^2=0<br>τ=0	-0.47 (-2.07, 1.13)<br>p=0.334	Dose: -0.03 (p=0.820)<br>Intervention (Synbiotic vs. Probiotic): -0.29 (p=0.672)<br>Operation (Hepatobiliary vs. Gut): -0.29 (p=0.672)<br>ROB (Low vs. High): -0.29 (p=0.672)<br>Duration: 0.01 (p=0.855)<br>Timing (Post vs. Peri) : -0.29 (p=0.672)
Anastomotic leakage	0.73<br>(0.43, 1.24)	-1.17<br>p=0.244	Komatsu, 2016<br>Kotzampassi, 2015<br>Zhang, 2012<br>Yokoyama, 2014<br>Yang, 2016<br>Tanaka, 2012<br>Sadahiro, 2014<br>Rayes, 2007<br>Mizuta, 2016	Q=8.31<br>p=0.404 (df=8)<br>I^2=3.69	τ^2=0.027<br>τ=0.163	-1.38 (-2.97, 0.22)<br>p=0.080	Dose: 0.27 (p=0.484)<br>Intervention (Synbiotic vs. Probiotic): 0.95 (p=0.082)<br>Operation (Hepatobiliary vs. Gut): 0.11 (p=0.899)<br>ROB (Low vs. High): -0.29 (p=0.640)<br>Duration: 0.01 (p=0.889)<br>Timing (Pre vs. Peri): -1.32 (p=0.400)
Deep organ space	0.62<br>(0.31, 1.25)	-1.34<br>p=0.180	Aisu, 2015<br>Komatsu,2016<br>Okazaki, 2013<br>Sadahiro, 2014<br>Mizuta, 2016	Q=0.553<br>p=0.968 (df=4)<br>I^2=0	τ^2=0<br>τ=0	-1.83 (-2.85, -0.81)<br>p=0.011	Dose: NOT ESTIMABLE<br>Intervention (Synbiotic vs. Probiotic): -0.19 (p=0.799)<br>Operation (Mixed vs. Gut): 0.78 (p=0.509)<br>ROB (Low vs. High): -0.38 (p=0.598)<br>Duration: 0.001 (p=0.992)<br>Timing: NOT ESTIMABLE
Diarrhea	0.49<br>(0.37, 0.67)	-4.59<br>p=0.000004	Diepenhorst, 2011<br>Liu, 2010<br>Liu, 2013<br>Liu, 2015<br>Yang, 2016<br>Tanaka, 2012<br>Zhang, 2013	Q=3.61<br>p=0.730 (df=6)<br>I^2=0	τ^2=0<br>τ=0	-0.27 (-1.92, 1.38)<br>p=0.691	Dose: 0.05 (p=0.688)<br>Intervention (Synbiotic vs. Probiotic): -0.29 (p=0.714)<br>Operation (Hepatobiliary vs. Gut): -0.76 (p=0.438)<br>ROB (Low vs. High): -0.32 (p=0.659)<br>Duration: -0.09 (p=0.223)<br>Timing (Post vs. Peri): 1.39 (p=0.250)
Intraabdominal abscess	0.69<br>(0.35, 1.37)	-1.07<br>p=0.285	Flesh, 2017<br>Kanazawa, 2005<br>Zhang, 2012<br>Yokoyama, 2014<br>Usami, 2011<br>Rayes, 2012<br>Sugawara, 2006<br>Yokoyama, 2016	Q=5.07<br>p=0.651 (df=7)<br>I^2=0	τ^2=0<br>τ=0	0.31 (-1.97, 2.59)<br>p=0.749	Dose: -0.004 (p=0.993)<br>Intervention (Synbiotic vs. Probiotic): -1.17 (p=0.350)<br>Operation (Hepatobiliary vs. Gut): -0.66 (p=0.457)<br>ROB (Low vs. High): -0.82 (p=0.356)<br>Duration: -0.04 (p=0.381)<br>Timing (Post vs. Peri): -0.06 (p=0.952), (Pre vs. Peri): 1.24 (p=0.334)

Mortality	1.17<br>(0.54, 2.57)	0.40<br>p=0.691	Anderson, 2003<br>Eguchi, 2011<br>Mangell, 2012<br>McNaught, 2002<br>Nomura, 2007<br>Sommacal, 2015<br>Rayes, 2007	Q=7.54<br>p=0.274 (df=6)<br>I^2=20.4	τ^2=0.226<br>τ=0.476	-2.03 (-4.15, 0.10)<br>p=0.058	Dose: -1.10 (p=0.327)<br>Intervention (Synbiotic vs. Probiotic): -0.31 (p=0.745)<br>Operation (Hepatoiliary vs. Gut): -0.67 (p=0.487)<br>ROB (Low vs. High): 0.54 (p=0.557)<br>Duration: 0.20 (p=0.340)<br>Timing: NOT ESTIMABLE
MRSA infection	0.34<br>(0.11, 1.06)	-1.87<br>p=0.062	Eguchi, 2011<br>Komatsu, 2016<br>Kotzampassi, 2015<br>Usami, 2011	Q=0.891<br>p=0.828 (df=3)<br>I^2=0	τ^2=0<br>τ=0	-1.97 (-2.96, -0.97)<br>p=0.014	Dose: NOT ESTIMABLE<br>Intervention (Synbiotic vs. Probiotic): 0.09 (p=0.944)<br>Operation (Hepatobiliary vs. Gut): -1.06 (p=0.402)<br>ROB (Low vs. High): -1.02 (p=0.526)<br>Duration: -0.08 (p=0.540)<br>Timing: NOT ESTIMABLE
Peritonitis	0.34<br>(0.09, 1.32)	-1.56<br>p=0.119	Zhang, 2012<br>Rayes, 2007<br>Zhang, 2013<br>Grąt, 2017	Q=1.75<br>p=0.625 (df=3)<br>I^2=0	τ^2=0<br>τ=0	-3.67 (-21.8, 14.5)<br>p=0.475	Dose: -0.57 (p=0.422)<br>Intervention (Synbiotic vs. Probiotic): -1.67 (p=0.229)<br>Operation (Hepatobiliary vs. Gut): -0.56 (p=0.699)<br>ROB (Low vs. High): -1.67 (p=0.229)<br>Duration: NOT ESTIMABLE<br>Timing: NOT ESTIMABLE
Pneumonia	0.55<br>(0.38, 0.80)	-3.12<br>p=0.002	Aisu, 2015<br>Flesh, 2017<br>Kanazawa, 2005<br>Kotzampassi, 2015<br>Liu, 2010<br>Liu, 2013<br>Liu, 2015<br>Okazaki, 2013<br>Zhang, 2012<br>Yokoyama, 2014<br>Yang, 2016<br>Tanaka, 2012<br>Reddy, 2007<br>Rayes, 2007<br>Rayes, 2005<br>Rayes, 2002a<br>Rayes, 2002<br>Rayes, 2012<br>Rammohan, 2015<br>Zhang, 2013<br>Sugawara, 2006<br>Yokoyama, 2016	Q=15.7<br>p=0.789 (df=21)<br>I^2=0	τ^2=0<br>τ=0	-0.47 (-1.49, 0.56)<br>p=0.355	Dose: 0.08 (p=0.665)<br>Intervention (Synbiotic vs. Probiotic): 0.31 (p=0.422)<br>Operation (Hepatobiliary vs. Gut): -0.19 (p=0.687), (Mixed vs. Gut): 0.84 (p=0.285)<br>ROB (Low vs. High): -0.16 (p=0.687)<br>Duration: 0.01 (p=0.775)<br>Timing (Post vs. Peri): -0.14 (p=0.834), (Pre vs. Peri): -0.45 (p=0.583)
Reoperation	0.43<br>(0.14, 1.29)	-1.51<br>p=0.132	Komatsu, 2016<br>Mangell, 2012<br>Grąt, 2017	Q=1.22<br>p=0.545 (df=2)<br>I^2=0	τ^2=0<br>τ=0	0.57 (-28.5, 29.6)<br>p=0.845	NOT ESTIMABLE

Sepsis	0.69<br>(0.58, 0.81)	-4.56<br>p=0.00001	Eguchi, 2011<br>Liu, 2013<br>Liu, 2015<br>Zhang, 2012<br>Rayes, 2007<br>Rayes, 2002<br>Rammohan, 2015	Q=5.43<br>p=0.491 (df=6)<br>I^2=0	τ^2=0<br>τ=0	-0.97 (-1.67, -0.26)<br>p=0.017	Dose: 0.55 (p=0.062)<br>Intervention (Synbiotic vs. Probiotic): -1.12 (p=0.154)<br>Operation (Hepatobiliary vs. Gut): -1.23 (p=0.168), (Mixed vs. Gut): -0.73 (p=0.650)<br>ROB (Low vs. High): -1.12 (p=0.154)<br>Duration: 0.12 (p=0.065)<br>Timing (Post vs. Peri): -0.73 (p=0.650), (Pre vs. Peri): -1.71 (p=0.097)
SSI	0.55<br>(0.39, 0.78)	-3.36<br>p=0.001	Flesh,2017<br>Kanazawa, 2005<br>Komatsu, 2016<br>Kotzampassi, 2015<br>Okazaki, 2013<br>Zhang, 2012<br>Zhang, 2013<br>Sugawara, 2006<br>Yokoyama, 2016<br>Mizuta, 2016	Q=9.77<br>p=0.369 (df=9)<br>I^2=7.89	τ^2=0.027<br>τ=0.163	-1.24 (-2.37, -0.11)<br>p=0.035	Dose: 0.40 (p=0.136)<br>Intervention (Synbiotic vs. Probiotic): 0.22 (p=0.592)<br>Operation (Hepatobiliary vs. Gut): 0.0001 (p=1.0), (Mixed vs. Gut): -1.09 (p=0.326)<br>ROB (Low vs. High): -0.21 (p=0.582)<br>Duration: -0.03 (p=0.403)<br>Timing (Post vs. Peri): -0.06 (p=-0.913), (Pre vs. Peri): -0.71 (p=0.537)
Superficial incisional	0.53<br>(0.38, 0.74)	-3.77<br>p=0.0002	Aisu, 2015<br>Komatsu, 2016<br>Liu, 2010<br>Liu, 2015<br>Okazaki, 2013<br>Yokoyama, 2014<br>Yang, 2016<br>Usami, 2011<br>Tanaka, 2012<br>Sadahiro, 2014<br>Reddy, 2007<br>Rayes, 2007<br>Rayes, 2005<br>Rammohan, 2015<br>Mizuta, 2016	Q=6.65<br>p=0.947 (df=14)<br>I^2=0	τ^2=0<br>τ=0	-0.17 (-1.08, 0.75)<br>p=0.703	Dose: 0.28 (p=0.240)<br>Intervention (Synbiotic vs. Probiotic): 0.14 (p=0.670)<br>Operation (Hepatobiliary vs. Gut): -0.19 (p=0.674), (Mixed vs. Gut): -1.44 (p=0.335)<br>ROB (Low vs. High): -0.2603 (p=0.441)<br>Duration: -0.03 (p=0.338)<br>Timing (Post vs. Peri): -0.46 (p=0.778), (Pre vs. Peri): 0.33 (p=0.706)
UTI	0.32<br>(0.18, 0.57)	-3.89<br>p=0.0001	Aisu, 2015<br>Eguchi, 2011<br>Kotzampassi. 2015<br>Liu, 2010<br>Liu, 2013<br>Liu, 2015<br>Okazaki, 2013<br>Yang, 2016<br>Rayes, 2007<br>Rayes, 2005<br>Rayes, 2002a<br>Rayes, 2002<br>Rammohan, 2015<br>Zhang, 2013	Q=9.51<br>p=0.734 (df=13)<br>I^2=0	τ^2=0<br>τ=0	-0.17 (-1.84, 1.51)<br>p=0.832	Dose: -0.53 (p=0.104)<br>Intervention (Synbiotic vs. Probiotic): -0.22 (p=0.722)<br>Operation (Hepatobiliary vs. Gut): -0.48 (p=0.454), (Mixed vs. Gut): 1.01 (p=0.399)<br>ROB (Low vs. High): -0.19 (p=0.746)<br>Duration: -0.17 (p=0.071)<br>Timing (Post vs. Peri): -0.27 (p=0.700)

† Egger's regression intercept test for asymmetry of the funnel plots; Dose – dose of probiotic (log), ROB – risk of bias, Post – post operation, Pre – pre operation, Peri – peri operation, SSI-surgical site infection

Outcome	SMD (95%CI)	Z-value	References	Heterogeneity	Tau	Intercept (95%CI)†	Meta-regression coefficients
Blood loss	0.004 (-0.120, 0.128)	0.059 p=0.953	Komatsu, 2016<br>Liu, 2010<br>Liu, 2015<br>Yang, 2016<br>Sadahiro, 2014<br>Rammohan, 2015<br>Sugawara, 2006	Q=3.15<br>p=0.789 (df=6)<br>I^2=0	τ^2=0<br>τ=0	-0.43 (-2.92, 2.06)<br>p=0.675	Dose: -0.04 (p=0.642)<br>Intervention (Synbiotic vs. Probiotic): -0.01 (p=0.922)<br>Operation (Hepatobiliary vs. Gut): 0.16 (p=0.373)<br>ROB (Low vs. High): -0.16 (p=0.373)<br>Duration: -0.02 (p=0.135)<br>Timing: NOT ESTIMABLE
Duration of antibiotic therapy	-0.60 (-1.09, -0.10)	-2.36 p=0.018	Kanazawa, 2005<br>Liu, 2010<br>Liu, 2013<br>Liu, 2015<br>Yang, 2016<br>Tanaka, 2012<br>Sommacal, 2015<br>Rayes, 2002a<br>Zhang, 2013<br>Sugawara, 2006	Q=102<br>p<0.00001 (df=9)<br>I^2=91	τ^2=0.575<br>τ=0.758	-4.91 (-15.57, 5.74)<br>p=0.319	Dose: -0.16 (p=0.514)<br>Intervention (Synbiotic vs. Probiotic) : -0.66 (p=0.212)<br>Operation (Hepatobiliary vs. Gut): -0.86 (p=0.085)<br>ROB (Low vs. High): -0.50 (p=0.342)<br>Duration: 0.08 (p=0.085)<br>Timing (Post vs. Peri): -0.95 (p=0.083)
Duration of postoperative pyrexia	-0.44 (-0.68, -0.192	-3.50 p=0.0005	Liu, 2010<br>Liu, 2013<br>Liu, 2015<br>Yang, 2016	Q=4.85<br>p=0.183 (df=3)<br>I^2=38.1	τ^2=0.024<br>τ=0.154	2.90 (-15.65, 21.46)<br>p=0.570	Dose: -0.17 (p=0.072)<br>Intervention: NOT ESTIMABLE<br>Operation: NOT ESTIMABLE<br>ROB: NOT ESTIMABLE<br>Duration: -0.13 (p=0.072)<br>Timing: NOT ESTIMABLE
Fluid diet	-0.35 (-0.58, -0.12)	-3.00 p=0.003	Liu, 2010<br>Liu, 2015<br>Yang, 2016	Q=1.31<br>p=0.520 (df=2)<br>I^2=0	τ^2=0<br>τ=0	-0.06 (-52.91, 52.80)<br>p=0.991	Dose: NOT ESTIMABLE<br>Intervention: NOT ESTIMABLE<br>Operation: NOT ESTIMABLE<br>ROB: NOT ESTIMABLE<br>Duration: NOT ESTIMABLE<br>Timing: NOT ESTIMABLE
Hospital stay	-0.48 (-0.66, -0.30)	-5.17 p=0.0000002	Kanazawa, 2005<br>Kotzampassi, 2015<br>Liu, 2010<br>Liu, 2015<br>Zhang, 2012<br>Yang, 2016<br>Usami, 2011<br>Sommacal, 2015<br>Rayes, 2002a<br>Zhang, 2013	Q=20.2<br>p=0.042 (df=11)<br>I^2=45.7	τ^2=0.045<br>τ=0.213	0.60 (-3.87, 5.07)<br>p=0.771	Dose: -0.03 (p=0.773)<br>Intervention (Synbiotic vs. Probiotic): -0.25 (p=0.183)<br>Operation (Hepatobiliary vs. Gut): -0.25 (p=0.183)<br>ROB (Low vs. High): -0.002 (p=0.994)<br>Duration: -0.005 (p=0.7434)<br>Timing (Post vs. Peri): -0.10 (p=0.679), (Pre vs. Peri): -0.23 (p=0.551)

			Sugawara, 2006<br>Mizuta, 2016				
ICU stay	-0.10<br>(-0.36, 0.16)	-0.73<br>p=0.460	Kanazawa, 2005<br>Usami, 2011<br>Tanaka, 2012<br>Rayes, 2002a	Q=1.35<br>p=0.719 (df=3)<br>I^2=0	τ^2=0<br>τ=0	3.23 (-19.10, 25.56)<br>p=0.597	Dose: -0.05 (p=0.922)<br>Intervention: NOT ESTIMABLE<br>Operation (Hepatobiliary vs. Gut): 0.25 (p=0.406)<br>ROB (Low vs. High): -0.28 (p=0.357)<br>Duration: 0.009 (p=0.697)<br>Timing (Post vs. Peri): -0.02 (p=0.944)
Operating time	0.02<br>(-0.14, 0.18)	0.26<br>p=0.797	Komatsu, 2016<br>Liu, 2010<br>Liu, 2013<br>Liu, 2015<br>Zhang, 2012<br>Yang, 2016<br>Sadahiro, 2014<br>Rayes, 2002a<br>Zhang, 2013<br>Sugawara, 2006<br>Mizuta, 2016	Q=19.6<br>p=0.033<br>(df=10)<br>I^2=49.0	τ^2=0.034<br>τ=0.184	0.20 (-3.07, 3.47)<br>p=0.893	Dose: -0.06 (p=0.502)<br>Intervention: -0.04 (p=0.820<br>Operation (Hepatobiliary vs. Gut): 0.06 (p=0.754)<br>ROB (Low vs. High): 0.005 (p=0.979)<br>Duration: -0.0006 (p=0.966)<br>Timing (Post vs. Peri): 0.28 (p=0.248), (Pre vs. Peri): -0.15 (0.665)
Solid diet	-0.31<br>(-0.50, -0.12)	-3.26<br>p=0.001	Aisu, 2015<br>Liu, 2010<br>Liu, 2015<br>Yang, 2016	Q=1.97<br>p=0.578 (df=3)<br>I^2=0	τ^2=0<br>τ=0	2.44 (-8.04, 12.93)<br>p=0.422	Dose: NOT ESTIMABLE<br>Intervention: NOT ESTIMABLE<br>Operation: NOT ESTIMABLE<br>ROB (Low vs. High): -0.26 (p=0.194)<br>Duration: NOT ESTIMABLE<br>Timing: NOT ESTIMABLE

† Egger's regression intercept test for asymmetry of the funnel plots; Dose – dose of probiotic (log), ROB – risk of bias, Post – post operation, Pre – pre operation, Peri – peri operation

Table S2. Risk of bias assesement.

Study reference	Publication year	Random sequence generation (selection bias)	Allocation concealment (selection bias)	Blinding of participants and personnel (performance bias)	Blinding of outcome assessment (detection bias)	Incomplete outcome data addressed (attrition bias)	Selective reporting (reporting bias)	Other bias	No. of low assesments
Aisu	2015	H	H	H	H	L	L	?	2
Anderson	2003	L	L	L	L	L	?	?	5
Diepenhorst	2011	L	L	?	?	?	L	?	3
Eguchi	2011	?	?	?	?	L	?	?	1
Flesh	2017	L	H	L	H	?	H	?	2
Grąt	2017	L	L	L	L	L	L	?	6
Horvat	2010	?	?	L	L	H	?	L	3
Kanazawa	2005	?	?	?	L	H	?	?	1
Komatsu	2016	L	L	H	H	L	L	L	5
kotzampassi	2015	?	L	L	L	L	L	H	5
Liu	2011	L	L	H	L	H	H	L	4
Liu	2013	L	L	L	L	H	?	L	5
Liu	2015	L	L	L	L	?	L	?	5
Mangell	2012	?	?	L	L	H	L	L	4
McNaught	2002	L	?	H	?	H	?	?	1
Mizuta	2016	L	H	?	?	L	?	?	2
Nomura	2007	?	?	?	?	L	?	?	1
Okazaki	2013	?	?	H	?	L	?	H	1
Rammohan	2015	L	H	H	H	L	L	?	3
Rayes	2007	?	?	L	L	H	H	?	2
Rayes	2005	?	?	L	L	L	H	?	3
Rayes	2002a	?	?	?	?	H	H	?	0
Rayes	2002	?	?	?	?	H	H	?	0
Rayes	2012	?	?	L	L	H	H	?	2

Reddy	2007	?	?	?	?	L	?	?	1
Sadahiro	2014	L	L	L	L	L	L	?	6
Sommacal	2015	L	L	L	L	L	L	L	7
Sugawara	2006	?	H	?	?	L	L	?	2
Tanaka	2012	L	H	H	H	L	L	?	3
Usami	2011	L	L	L	?	L	H	H	4
Yang	2016	L	L	L	L	H	L	?	5
Yokoyama	2014	L	L	H	L	L	L	H	5
Yokoyama	2016	L	H	H	H	L	?	?	2
Zhang	2012	?	L	L	L	L	?	L	5
Zhang	2013	H	H	H	H	L	L	?	2

L-low risk of bias, H-high risk of bias, ?-unclear risk of bias
